# COL6A3 Exosomes Promote Tumor Dissemination and Metastasis in Epithelial Ovarian Cancer

**DOI:** 10.3390/ijms25158121

**Published:** 2024-07-25

**Authors:** Chih-Ming Ho, Ting-Lin Yen, Tzu-Hao Chang, Shih-Hung Huang

**Affiliations:** 1 Gynecologic Cancer Center, Department of Obstetrics and Gynecology, Cathay General Hospital, Taipei 106, Taiwan; 2 School of Medicine, Fu Jen Catholic University, Hsinchuang, New Taipei City 242, Taiwan; 3 Department of Medical Research, Cathay General Hospital, Sijhih, New Taipei City 221, Taiwan; d119096015@tmu.edu.tw; 4 School of Medicine, Taipei Medical University, Taipei 110, Taiwan; 5 Graduate Institute of Biomedical Informatics, Taipei Medical University, Taipei 110, Taiwan; kevinchang@tmu.edu.tw; 6 Department of Pathology, Cathay General Hospital, Taipei 106, Taiwan; a68@cgh.org.tw

**Keywords:** exosomes, COL6A3, metastasis, epithelial ovarian cancer, aggressiveness, exosome inhibitor

## Abstract

Our study explores the role of cancer-derived extracellular exosomes (EXs), particularly focusing on collagen alpha-3 (VI; COL6A3), in facilitating tumor dissemination and metastasis in epithelial ovarian cancer (EOC). We found that COL6A3 is expressed in aggressive ES2 derivatives, SKOV3 overexpressing COL6A3 (SKOV3/COL6A3), and mesenchymal-type ovarian carcinoma stromal progenitor cells (MSC-OCSPCs), as well as their EXs, but not in less aggressive SKOV3 cells or ES2 cells with COL6A3 knockdown (ES2/shCOL6A3). High COL6A3 expression correlates with worse overall survival among EOC patients, as evidenced by TCGA and GEO data analysis. In vitro experiments showed that EXs from MSC-OCSPCs or SKOV3/COL6A3 cells significantly enhance invasion ability in ES2 or SKOV3/COL6A3 cells, respectively (both, *p* <0.001). In contrast, ES2 cells with ES2/shCOL6A3 EXs exhibited reduced invasion ability (*p* < 0.001). In vivo, the average disseminated tumor numbers in the peritoneal cavity were significantly greater in mice receiving intraperitoneally injected SKOV3/COL6A3 cells than in SKOV3 cells (*p* < 0.001). Furthermore, mice intravenously (IV) injected with SKOV3/COL6A3 cells and SKOV3/COL6A3-EXs showed increased lung colonization compared to mice injected with SKOV3 cells and PBS (*p* = 0.007) or SKOV3/COL6A3 cells and PBS (*p* = 0.039). Knockdown of COL6A3 or treatment with EX inhibitor GW4869 or rapamycin-abolished COL6A3-EXs may suppress the aggressiveness of EOC.

## 1. Introduction

Ovarian cancer has the highest mortality rate among gynecological cancers [1]. Due to the lack of obvious symptoms and effective screening methods, most patients are diagnosed at an advanced stage, and surgery cannot remove all tumors, resulting in a poor prognosis [2]. Advanced epithelial ovarian cancer (EOC) disseminates widely in the abdominal cavity, featuring residual tumors after debulking surgery and resistance to chemotherapy drugs [3,4,5]. Poor prognostic factors affect overall survival in advanced-stage EOC patients; such factors include suboptimal debulking surgery, chemotherapy resistance, massive ascites, histologic clear cell or mucinous carcinoma, and stage IV diseases. EOC metastasis mainly occurs through peritoneal dissemination. At least one-third of patients with EOC develop ascites with a poor prognosis, in which their five-year survival declines sharply [6].

Extracellular vesicles (EVs) play crucial roles in intercellular communication, tumor progression and metastasis, immune modulation, drug resistance, and angiogenesis. The roles of extracellular vesicles in ovarian cancer have been studied in relation to chemoresistance, tumor microenvironment modulation, biomarkers for diagnosis and prognosis, metastasis, and immune evasion. Recent studies have indicated that cancer cells secrete extracellular vesicles (EVs), promoting cancer invasion, dissemination, and the development of the cancer microenvironment [7]. EVs include exosomes (EXs) and microvesicles, which are small membrane vesicles containing microRNAs (miRNAs), messenger RNAs, and proteins [8,9]. EXs are small vesicles, ~30–150 nm in size, that develop within endosomes through membrane invaginations [8]. The cancer-derived EXs participate in the promotion of dissemination and metastasis from the initial stages to the development of secondary tumors [7,10,11,12]. EXs possess several unique advantages as biomarkers for the early detection of dissemination because they are stable, abundant, and tumor-specific and can be detected in the blood or ascites [9]. Recent evidence has demonstrated that cancer EXs exert both autocrine and paracrine effects on the microenvironment [13]. A previous study indicated that ovarian cancer EXs could transfer CD44 to the peritoneal mesothelium to invade the physical barrier [14]. Ascites-derived EXs from ovarian cancer patients carry MMP1 mRNA and induce apoptosis in mesothelial cells [14]. EXs released from EOC cells promote and shift the conversion from normal fibroblasts and adipose-derived mesenchymal stem cells to cancer-associated fibroblasts (CAFs) [15,16] and activate mesenchymal phenotypes [17]. EOC primary tumors can create an advantageous microenvironment to help tumors attach in distant organs through malignant ascites-derived EXs, which are dynamically remodeling tumor stroma that form metastatic niches in the omentum from CAF conversion [18]. 

Collagen type II secreted from stromal fibroblasts may promote tumor growth and angiogenesis [19], while collagen type VI secreted from the base membrane directly affects tumor growth, invasion, and metastasis in various neoplasms [20]. Our previous study demonstrated that upregulation of collagen type VI α3 (COL6A3) may promote tumor invasion and metastasis in EOC [21]. The invasiveness was enhanced up to 10-fold when 25 μg of COL6 protein was added to SKOV3 cells [21]. Furthermore, COL6A3 has been reported to be associated with cisplatin resistance in an autocrine manner [22]. A recent study disclosed that chemotherapy upregulated the expression of collagen type VI in the omentum and peritoneum in EOC patients [23]. COL6 is primarily derived from tumor stroma, and increased COL6 gene expression in solid tumors is associated with shortened progression-free intervals and survival [24]. 

The EXs from more aggressive EOC cells strongly enhance aggressive behavior in less aggressive tumors, which promotes aggressiveness without changing the properties of EOC cells from non-aggressive to aggressive [14]. To further investigate which components mainly affect the aggressiveness underlying EXs from more aggressive ovarian cancer cells and the microenvironment of ascites, we used the ES2 cell line that was isolated from the ovary of a female human with clear cell carcinoma as a more aggressive phenotype exhibiting a fibroblast-like morphology and the SKOV3 cell line that was derived from the ascites of a female human with serous cystadenocarcinoma as a less aggressive phenotype exhibiting an epithelial-like morphology. Our recent study showed COL6A3 could be detected in culture media and is abundant in primary ovarian cancer tissues, disseminated metastatic omentum tissues, EOC spheroids, and MSC-OCSPCs, which appear to possess the new function of promoting EOC in EMT, stemness, tumor growth, and metastasis [21]. COL6A3 belongs to an extracellular matrix (ECM) gene and is classified as a mesenchymal-type-associated gene, which was determined to be the worst prognosis subtype in EOC via TCGA molecular subtype analyses [21]. 

In this study, we elucidated for the first time COL6A3 transport via EXs from EOC tissues and MSC-OCSPCs conferring invasiveness and metastasis in EOC cells. We evaluated treatment strategies focusing on lysosomes, autophagy inhibition, and possible target genes in EOC cells and EXs in in vitro experiments and in vivo live mouse models.

## 2. Results

### 2.1. Characterization of Exosomes in EOC and Ascites-Derived Cell Lines

**Figure 1:**
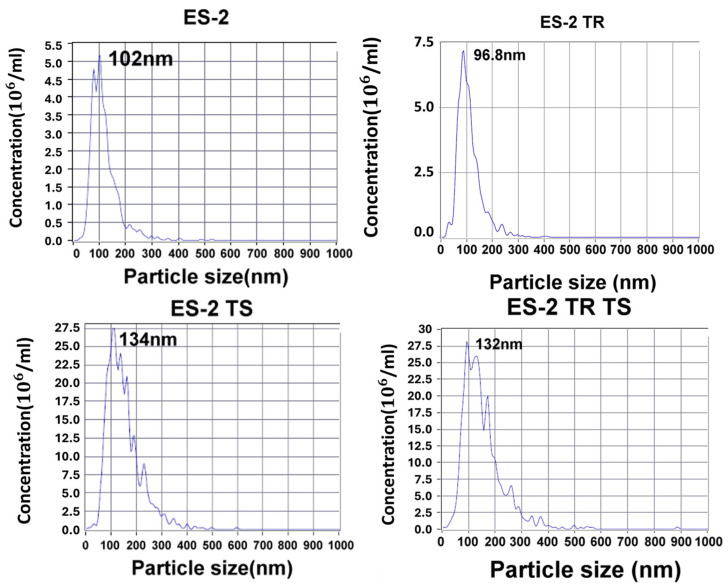
Characteristics of EV nanoparticle-tracking analyses of the particle sizes of ES2 EVs, ES2TR EVs, ES2 tumorsphere EVs, and ES2TR tumorsphere EVs. The vertical axes in the graphs show the number of EV particles (×10^6^)/mL, and the horizontal axes indicate the particle size (nm).

We first characterized the particle sizes of exosomes (EXs) from more aggressive ES2 cells, ES2 paclitaxel-resistant cells (ES2TR), ES2-derived tumor spheres (ES2 TS), and ES2 paclitaxel-resistant cells-derived tumor spheres (ES2TR TS), which were established in our lab as previously described [21]. Using nanoparticle-tracking analysis, the mean particle sizes of ES2 EXs, ES2TR EXs, ES2 TS EXs, and ES2TR TS EXs were determined to be 102 nm, 96.8 nm, 134 nm, and 132 nm, respectively. The mean concentrations of the number of particles (×10^6^/mL) of ES2 EXs, ES2TR EXs, ES2 TS EXs, and ES2TR TS EXs were 4.8, 7.3, 2.8, and 2.8, respectively (Figure 1). ES2 cells were maintained in a humidified atmosphere containing 5% CO_2_ at 37 °C and grown in McCoy’s 5A medium with 10% FBS. As previously described, we developed a paclitaxel-resistant ES2 cell line by continuously exposing cells to paclitaxel [25]. The final paclitaxel concentrations that induced paclitaxel-resistant subclones, called ES2TR, measured 160 nM. The ES2TR TS was developed using ES2TR cells cultured in DMEM/F12 medium with 20 ng/mL of bFGF, 20 ng/mL of EGF, 10 ng/mL of IGF, and 2% B27 (Invitrogen, Carlsbad, CA, USA). The dissociated single cells (1 × 10^5^ cells/mL) were seeded into ultra-low attachment plates (Corning 3262, Pittston, PA, USA). After 7 days, we counted the spheres formed with an Olympus light microscope (Olympus, Tokyo, Japan). Then, the tumor spheres obtained after 14 days were harvested and analyzed with flow cytometry. 

**Figure 2:**
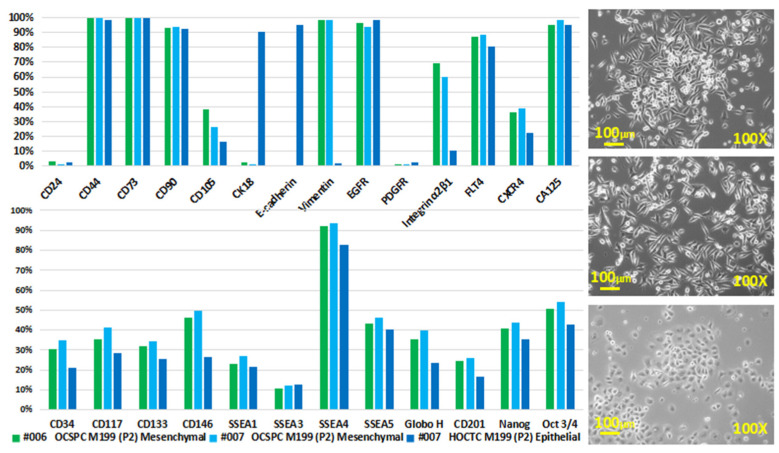
Exosome characterization of cell lines. (**right**) These are phase-contrast images of #006 and #007 human ovarian carcinoma ascites (**upper** and **middle**) and #007 human ovarian carcinoma tissue (**lower**)-derived cells (P2). The adherent culture conditions were M199 + 10% FBS + 20 ng/mL of EGF + 0.4 μg/mL of hydrocortisone. (**left**) These are surface expression markers of human ovarian carcinoma ascites and tissue-derived cells with spindle-like mesenchymal-like (MSC-) (**right upper** and **middle**) ovarian carcinoma stromal progenitor cells (OCSPCs) and roundish epithelial-like (epi-) (**right lower**) ovarian-carcinoma-tissue-derived cells from 2 advanced ovarian cancer patients. (**left**) High expressions of vimentin in MSC-OCSPCs and CK18 and E-cadherin in epi-OCSPCs were noted. High expression of CD44, CD73, CD90, FLT4, CA125, and SSEA4 was noted in both cells.

The percentages of positive staining in terms of pluripotent and drug-resistance-related factors were substantially higher in ES2 TS and ES2TR160 TS than in ES2 and ES2 TR160, respectively, as determined via flow cytometric analysis [26]. Two morphologically different adherent cell populations of ovarian cancer stromal progenitor cells (OCSPCs) from two EOC patients’ ascites and tissues were cultured and isolated in selective conditional media [27] (Figure 2, right panel). Epithelial-like OCSPCs (epi-OCSPCs) promoted tumorigenesis; in contrast, mesenchymal-like OCSPCs (MSC-OCSPCs) enhanced the migration, invasion, and spheroid aggregation of EOC [28]. High expression of vimentin with low expression of CK18 and E-cadherin in MSC-OCSPCs and, in contrast, low expression of vimentin with high expression of CK18 and E-cadherin in epi-OCSPCs were characteristic in the two types of cells (Figure 2, left upper panel). The high CD133, CD117, SSEA4, and CA125 expression in epi-OCSPCs and MSC-OCSPCs revealed that OCSPCs processed stemness characteristics and malignance changes (Figure 2, left lower panel). 

**Figure 3:**
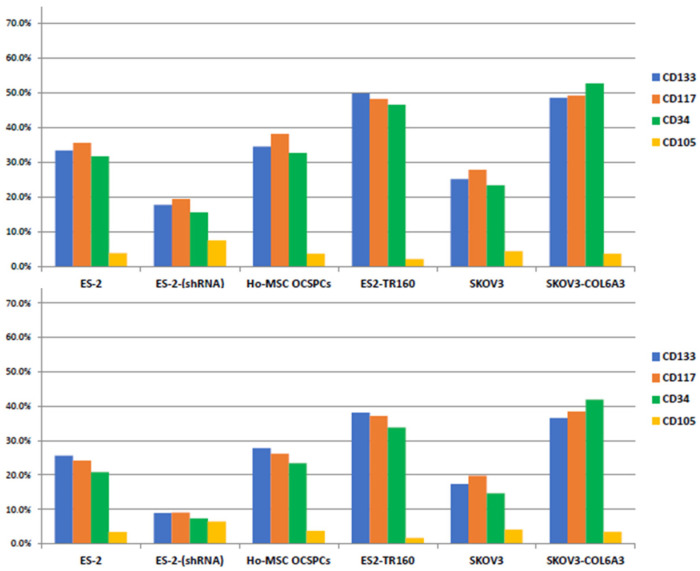
The percentage of positive stemness markers such as CD133, CD117, CD34, and CD105 was consistent in ES2, ES2-COL6A3 shRNA, MSC-OCSPCs, ES2TR160, SKOV3, and SKOV3-COL6A3 cells (**upper**) and exosomes (EXs) (**lower**). ES2TR160 and SKOV3-COL6A3 processed the highest percentage of CD133, CD117, and CD34 stemness phenotypes in cells and exosomes.

We further characterized the particle sizes of ascites-derived EXs using nanoparticle-tracking analysis. The mean particle sizes of the ascites-derived EXs from two advanced EOC patients were 100 nm and 94 nm, respectively. The mean concentrations of ascites-derived EX particles (×10^10^/mL) were 4.5 and 3.93, respectively, and the mentioned ascites cases were abundant in EX particles. The consistent expression of positive stemness surface expression markers of CD133, CD117, and CD34, as determined via flow cytometry in EOC cells and MSC-OCSPCs, and those derived from EXs revealed that EOC cells and MSC-OCSPC-derived EXs processed stemness characteristics as well as EOC cells and MSC-OCSPCs (Figure 3).

### 2.2. Invasion Ability of EOC-Cell-Line-Derived Exosomes

**Figure 4:**
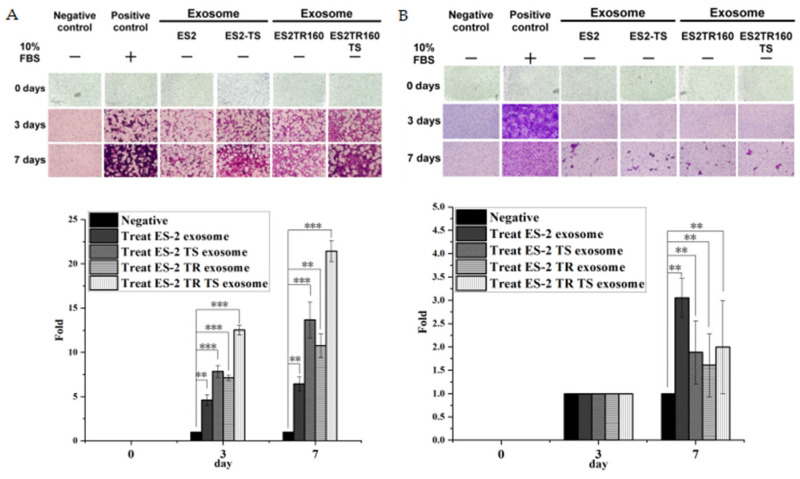
Invasion ability of EOC-cell-line-derived exosomes. Their invasion ability was examined in ES2 (**A**) and SKOV3 (**B**) treated with ES2, ES2TR, ES2 tumor sphere, and ES2TR tumor sphere exosomes and not treated with said exosomes. The invasion ability of exosomes from ES2, ES2 TS, ES2TR, and ES2TR TS was more remarkably enhanced in ES2 than in SKOV3 (*** *p* < 0.001 for ES2 and ** *p* < 0.01 for SKOV3, respectively).

We subsequently examined if EOC EXs promote EOC invasion. Our results indicated their invasion ability was significantly greater in ES2 cells treated with ES2 EXs, ES2TR160 EXs, ES2 TS EXs, or ES2TR160TS EXs than those that were not treated with EXs (*p* < 0.01 for ES2 EXs and ES2TR160 EXs and *p* < 0.001 for ES2 TS EXs and ES2TR160 TS EXs, respectively) (Figure 2). We next asked if different EOC cells and MSC-OCSPC-derived EXs processed different enhancements of invasion ability. We compared the invasion ability of EXs with degrees of aggressive EOC cell lines, including SKOV3 (serous type, less aggressive); ES2, ES2 TS, ES2TR160, and ES2TR TS (clear-cell-type-derived, more aggressive); and MSC-OCSPCs (obtained from advanced EOC patients with massive ascites cultured in selective conditional media). The invasion ability of ES2 was most enhanced by ES2TR TS EXs compared to ES2, ES2 TS, or ES2TR EXs (Figure 4A). We further asked if the EXs from more aggressive EOC cells have different degrees of invasiveness in more aggressive and less aggressive EOC cells. Our results revealed that the invasion ability of ES2, ES2 TS, ES2TR160, and ES2TR160 TS EXs was greater for ES2 (more aggressive) cells than for SKOV3 cells (less aggressive) (Figure 4B).

### 2.3. Invasion Ability of Autocrine and Paracrine Effects in EOC-Cell-Line-Derived Exosomes

**Figure 5:**
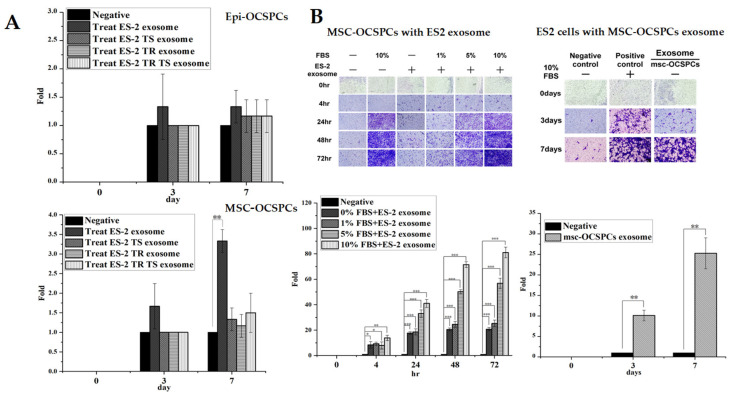
Invasion ability of autocrine and paracrine effects in EOC-cell-line-derived exosomes. (**A**) Invasion ability was examined in epi-OCSPCs and MSC-OCSPCs treated with ES2, ES2TR, ES2 tumor spheres, and ES2TR tumor sphere exosomes and not treated with said substances. The invasion ability was only significantly enhanced in the MSC-OCSPCs (** *p* < 0.01) treated with ES2 exosomes, not in epi-OCSPCs. (**B**) The invasion ability was substantially increased in the MSC-OCSPCs that were treated with ES2 exosomes than in those that were not (* *p* < 0.05; *** *p* < 0.001). Simultaneously, the invasion ability was greater in ES2 cells treated with MSC-OCSPC exosomes than in those without MSC-OCSPC exosomes (** *p* < 0.01).

We reasoned that the EOC EXs exert both autocrine and paracrine effects that enhance invasiveness for EOC cells and MSC-OCSPCs in the microenvironment. We asked which types of OCSPCs could be increased by highly aggressive EOC-cell-derived EXs. The results showed that only MSC-OCSPCs could be enhanced by ES2-derived EXs (*p* < 0.01) (Figure 5A). We next examined the paracrine effect of invasion ability in ES2 cells treated with MSC-OCSPC EXs or MSC-OCSPCs treated with ES2 EXs. The invasion ability was significantly greater in MSC-OCSPCs treated with ES2 EXs or ES2 cells treated with MSC-OCSPCs EXs than in those without EXs (*p* < 0.001, *p* < 0.01, respectively). (Figure 5B). This result implied that EXs from cancer cells or MSC-OCSPCs could reciprocally promote invasiveness through the paracrine effect.

### 2.4. Heat Map of Differential Expression of EOC Exosomes

**Figure 6:**
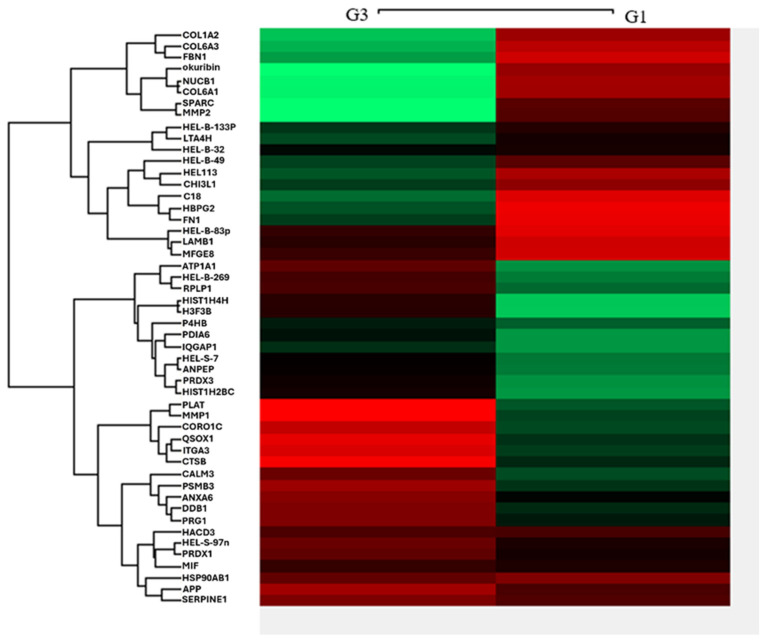
Heat map of differential protein expression of EOC exosomes. The heat map shows group 1—ES2 cells and ES2 EXs versus ES2 cells and group 3—MSC-OCSPCs and ES2 EXs versus MSC-OCSPCs, which were examined using LC-MS/MS analyses.

We further explored which components of EXs from more aggressive ES2 cells enhanced invasion in EOC cells and MSC-OCSPCs. Using LC-MS/MS analyses, we compared the differential expression levels >2 among different groups in group 1—ES2 cells and ES2 EXs versus ES2 cells; group 2—ES2 with ES2 TS EXs versus ES2 cells; and group 3—MSC-OCSPCs and ES2 EXs versus MSC-OCSPCs. The total number of differentially expressed genes in EOC exosomes that exhibited significant changes in expression levels was fifty. There were 26 upregulated and 24 downregulated DEGs in G3 and 27 upregulated and 23 downregulated DEGs in G1. The results for the heat map are shown in Figure 6. COL6A3 had one of the statistically differential expression levels between Log2 (group 1 versus group 3), with a value of 3.72, and Log2 (group 2 versus group 3), with a value of 3.07. Our previous study showed that collagen VI can accelerate tumor growth and metastasis in EOC [21]. Overexpression of COL6A3 in tumor cells can directly remodel their extracellular matrices (ECMs). This change in the ECM helps develop drug resistance and exacerbates metastasis in ovarian cancer [24]. Therefore, we speculated that COL6A3 transport through EXs may play a role in drug resistance and metastasis.

### 2.5. COL6A3 Expression in EOC Cell Lines and Derived Exosomes

**Figure 7:**
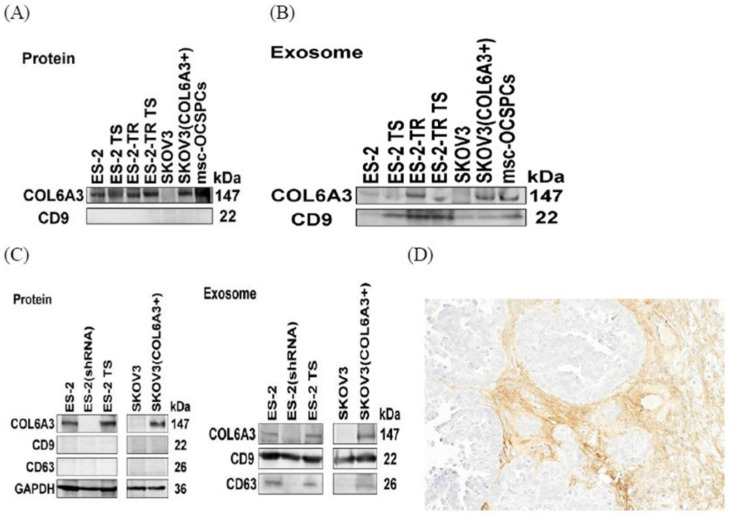
COL6A3 expression in EOC cell lines and derived exosomes. (**A**,**B**) COL6A3 was expressed in ES2 derivatives, SKOV3/COL6A3, and MSC-OCSPC-derived-exosomes and lysates, while there was no expression in SKOV3- and ES2/shRNA-derived exosomes and cell lysates. (**C**) The CD9 and CD63 representative exosome markers were seen in ES2-derivative-, SKOV3/COL6A3-, and MSC-OCSPCs-derived exosomes, but CD9 and CD63 were not detected in those cell lysates. (**D**) Immunostaining of COL6A3 was positive in ovarian serous carcinoma stromal cells, which surrounded cancer cells with negative staining.

We first checked the expression levels of collagen VI in more aggressive ES2, ES2TR, ES2 TS, and ES2TR TS and less aggressive SKOV3 cells and the EXs derived from them. Our results showed collagen VI (collagen alpha-3 (VI; COL6A3)) was present in more aggressive ES2, ES2TR, ES2 TS, and ES2TR TS cells and the EXs derived from ES2, ES2 TS, ES2TR, and ES2TR TS cells. In particular, the COL6A3 expressions were more prominent in ES2TR160- and ES2TR160-TS-derived EXs than in ES2- and ES2-TS-derived EXs, which suggested COL6A3 EXs might play a role in paclitaxel drug resistance. In contrast, collagen VI was absent in less aggressive SKOV3 cells and EXs; only a single cell line of this type was utilized (Figure 7A,B). We next overexpressed COL6A3 in less aggressive SKOV3 cells (SKOV3/COL6A3) and knocked down COL6A3 in more aggressive ES2 cells (ES2/shCOL6A3). The results showed that COL6A3 was expressed in ES2, ES2 TS, and SKOV3/COL6A3 cells and the EXs derived from them; in contrast, COL6A3 was not expressed in SKOV3 and ES2/shCOL6A3 cells and their EXs as determined via Western blot analysis (Figure 7C). This implies COL6A3 undergoes expression and secretion through the EX route in more aggressive EOC cells and EOC TS cells but is not expressed and secreted in less aggressive EOC cells or more aggressive EOC knockdown COL6A3 cells and EXs. We further verified whether the COL6A3 protein showed staining in ovarian tumor cells. COL6A3 showed strong staining in cancerous stromal cells but not in cancer cells of high-grade serous ovarian carcinoma paraffin-embedded tissue (as determined via immunohistochemistry) (Figure 7D). This result is consistent with the lack of expression of COL6A3 in less aggressive SKOV3 cells (serous type). Our previous work showed that the high expression of COL6A3 in an MSC-OCSPC culture medium enhanced the invasiveness of SKOV3, while knockdown of COL6A3 in MSC-OCSPCs inhibited the invasiveness of EOC and spheroids [21]. In this study, COL6A3 showed expression in more aggressive ES2, ES2 TS, ES2TR, ES2TR TS (clear-cell-type), and MSC-OCSPC cells and their EXs (Figure 7A,B). 

### 2.6. The Invasion Ability of Overexpressed and Knockdown COL6A3 Expression in EOC Cells with Those Exosomes

**Figure 8:**
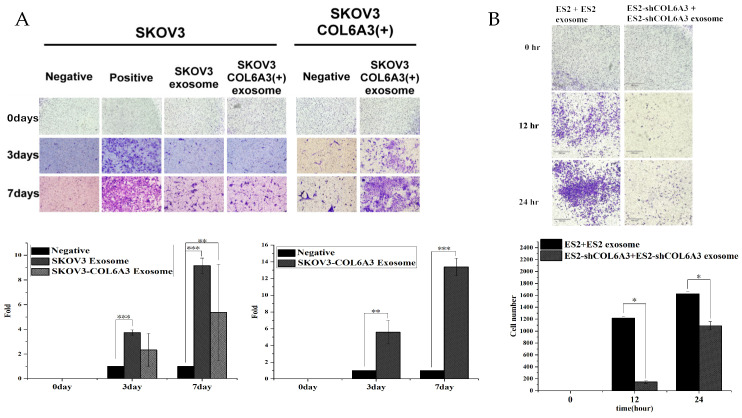
The invasion ability of overexpressed and knockdown EOC cells with the exosomes derived from them (**A**) was examined in SKOV3 and SKOV3-COL6A3 cells treated with and without these respective EXs. The invasion ability was significantly greater in SKOV3 and SKOV3-COL6A3 cells treated with the respective EXs than in those without EXs (** *p* < 0.01; *** *p* < 0.001). (**B**) Invasion ability was examined in ES2 cells with ES2 EXs and ES2 knockdown COL6A3 cells (ES2-shCOL6A3) with ES2-shCOL6A3 EXs. Invasion ability was significantly greater in ES2 cells with ES2 EXs than in ES2/shCOL6A3 cells with ES2-shCOL6A3 EXs (both, * *p* < 0.05).

We next examined the invasion ability of SKOV3 cells with or without SKOV3 EXs and SKOV3/COL6A3 cells with or without SKOV3/COL6A3 EXs. The invasion ability was significantly greater in SKOV3 cells with SKOV3 EXs and SKOV3/COL6A3 cells with SKOV3/COL6A3 EXs than in those without EXs (both *p* < 0.001). Moreover, the invasion ability was greater in SKOV3/COL6A3 EXs with SKOV3/COL6A3 cells than in SKOV3/COL6A3 EXs with SKOV3 cells (Figure 8A). Our results indicate that the extent to which invasion ability is enhanced by EOC-overexpressed COL6A3 EXs depends on varying degrees of aggressive malignant potential EOC cells. In contrast, invasion ability was significantly inhibited in ES2/shCOL6A3 cells with ES2/shCOL6A3 EXs compared to that in ES2 cells with ES2 EXs (*p* < 0.05) (Figure 8A). However, invasion ability was not significantly inhibited in ES2 cells with ES2/shCOL6A3 EXs when compared to that in ES2 cells with ES2 EXs (Figure 8B).

### 2.7. EOC-Derived EXs Accelerated Cancerous Peritoneal Dissemination

**Figure 9:**
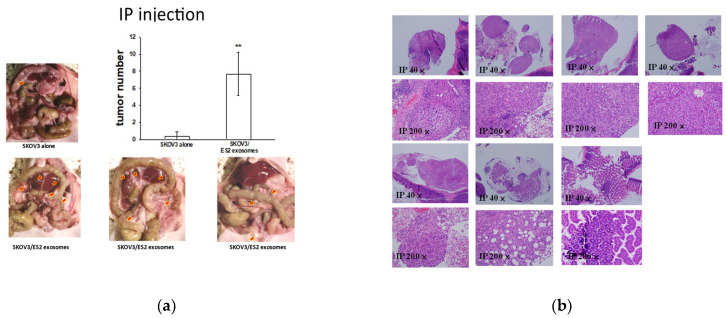
EOC-derived EXs accelerated cancerous peritoneal dissemination. (**a**) Representative pictures of 6/7 mice IP injected with 1 × 10^6^ SKOV3 cells with ES2 exosomes showing disseminated tumors (red arrows) in the peritoneal cavity compared to the 1/3 mice injected with 1 × 10^6^ SKOV3 cells with PBS (*p* = 0.097, as determined using Student’s *t*-test). The average disseminated tumor numbers in the peritoneal cavity were significantly greater in mice receiving SKOV3 cells with ES2-exosomes than in those administered SKOV3 cells with PBS (** *p* < 0.01, as determined using Student’s *t*-test). (**b**) Representative histologic pictures of disseminated peritoneal tumors are shown at microscopic scales of 40× and 200×.

We reasoned that EOC-derived EXs could accelerate cancerous peritoneal dissemination and lung colonization. The data indicated that the EXs from ES2 cells, a rapidly disseminated cell line, possessed greater invasion ability than the EXs from SKOV3 cells. Therefore, we examined whether ES2 EXs enhanced less aggressive EOC cells’ peritoneal dissemination in vivo. To this end, luciferase-expressing SKOV3 cells, which displayed a less aggressive phenotype, were injected into the peritoneal cavity, and 10 μg of EXs from more aggressive ES2 cells or phosphate-buffered saline (PBS) was intraperitoneally injected twice weekly for 6 weeks. In total, 6 of the 7 mice intraperitoneally injected with 1 × 10^6^ SKOV3 cells and ES2 EXs had a significantly greater disseminated burden in the peritoneal cavity compared with the 1 of 5 mice injected with 1 × 10^6^ SKOV3 cells and PBS (*p* = 0.023, as determined using Student’s *t*-test). The average disseminated tumor numbers in the peritoneal cavity were greater in the mice receiving SKOV3 cells with ES2 EXs than in those administered SKOV3 cells with PBS (*p* < 0.01, as determined via Student’s *t*-test) (Figure 9).

### 2.8. Overexpressed COL6A3 in EOC-Derived EXs Accelerated Cancerous Peritoneal Dissemination

**Figure 10:**
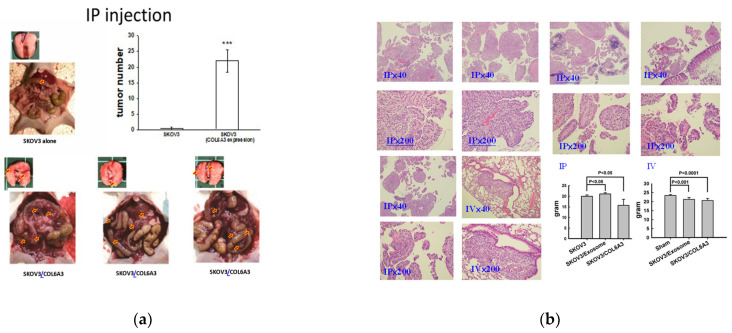
Overexpressed COL6A3 in EOC-derived EXs accelerated cancerous peritoneal dissemination. (**a**) Average disseminated tumor numbers in the peritoneal cavity were significantly greater in mice receiving SKOV3-overexpressed COL6A3 (SKOV3/COL6A3) than in SKOV3 cells (*** *p* < 0.001, as determined using Student’s *t*-test). Red arrows indicated disseminated tumors in the peritoneal cavity. A total of 1/8 of the mice IV injected with 1 × 10^6^ SKOV3/COL6A3 cells had colonization in the lung, while this was only the case for 0/32 of the mice injected with 1 × 10^6^ SKOV3 cells only (*p* = 0.043, as determined using Student’s *t*-test). (**b**) Representative histologic pictures of the peritoneal tumor and lung colonization are shown at microscopic scales of 40× and 200×. The right lower panel shows the differential body weights of mice among the IP and IV groups treated with SKOV3 cells, SKOV3 cells with ES2 exosomes, and SKOV3/COL6A3 cells.

Because COL6A3 exhibits expression in ES2 EXs, which might enhance invasiveness and dissemination, we next explored whether COL6A3 is a key element for enhancing dissemination from ES2 EXs. We overexpressed COL6A3 in SKOV3 cells (SKOV3/COL6A3) to see if COL6A3 accelerated peritoneal dissemination in vivo. To this end, 1 × 10^6^ SKOV3/COL6A3 cells, presumed to be more aggressive cells, or 1 × 10^6^ less aggressive SKOV3 cells were injected into the peritoneal cavity. Five of the six mice injected with 1 × 10^6^ SKOV3/COL6A3 cells had a greater disseminated burden in the peritoneal cavity than one of the five mice injected with 1 × 10^6^ SKOV3 cells (*p* = 0.036, as determined using Student’s *t*-test). The average disseminated tumor numbers in the peritoneal cavity were significantly greater in mice receiving SKOV3/COL6A3 cells than SKOV3 cells (*p* < 0.001, as determined using Student’s *t*-test) (Figure 10). 

### 2.9. Overexpressed COL6A3 in EOC-Derived EXs Accelerated Lung Colonization

**Figure 11:**
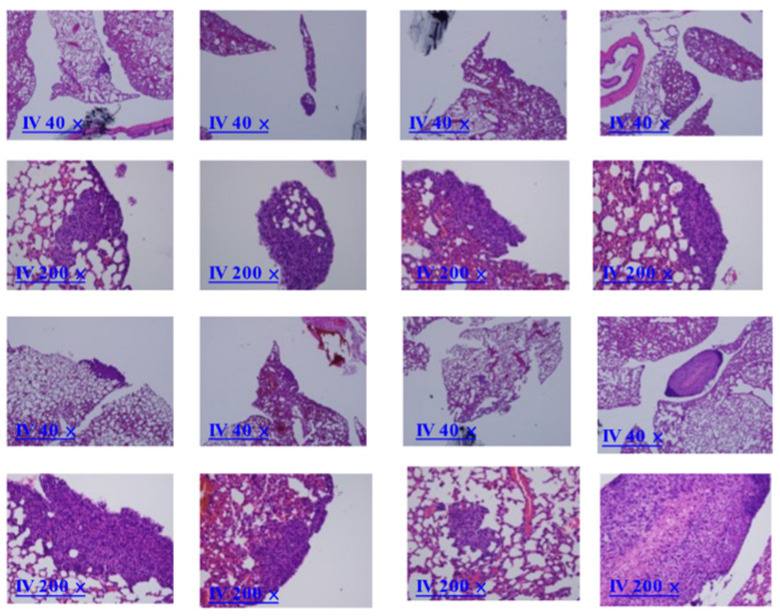
Overexpressed COL6A3 in EOC-derived EXs accelerated lung colonization. In total, 5/8 mice IV injected with 1 × 10^6^ SKOV3/COL6A3 cells and 10 μg of SKOV3/COL6A3 exosomes had colonization in the lung, while this was the case for 0/8 mice injected with 1 × 10^6^ SKOV3 cells and PBS (*p* = 0.007, as determined using Student’s *t*-test) and 1/8 mice IV injected with 1 × 10^6^ SKOV3/COL6A3 cells (*p* = 0.039) (Figure 10b). Histologic pictures of lung colonization tumors are shown at 40× and 200× microscope magnification.

We next examined whether SKOV3/COL6A3 cells also accelerated distant lung colonization in vivo. To this end, 1 × 10^6^ SKOV3/COL6A3 cells or 1 × 10^6^ SKOV3 cells were administered intravenously into the tail vein in mice. In total, 1 of the 8 mice intravenously injected with 1 × 10^6^ SKOV3/COL6A3 cells had colonization in the lung, while this was only the case for 0 of the 32 mice injected with 1 × 10^6^ SKOV3 cells (*p* = 0.043, as determined using Student’s *t*-test) (Figure 9). We next examined whether SKOV3/COL6A3 EXs help accelerate lung colonization in vivo. To this end, SKOV3/COL6A3 cells were intravenously injected with 10 μg of EXs from SKOV3/COL6A3 cells or phosphate-buffered saline (PBS) twice weekly for up to 10 weeks. A total of 5 of the 8 mice intravenously injected with 1 × 10^6^ SKOV3/COL6A3 cells and SKOV3/COL6A3 EXs had a significantly greater colonization burden in the lung compared with the 1 of 8 mice injected with 1 × 10^6^ SKOV3/COL6A3 cells (*p* = 0.039, as determined using Student’s *t*-test) (Figure 11). These data suggest that COL6A3 and EXs have more aggressive characteristics, which promote EOC cell dissemination and colonization in the peritoneal cavity and lung. In contrast, knockdown of COL6A3 in EOC spheroids inhibited COL6A3 expression in EOC spheroids, which decreased EOC spheroid formation, invasion, tumor growth, and metastasis [28].

### 2.10. The Overall Survival of COL6A3 Expression

**Figure 12:**
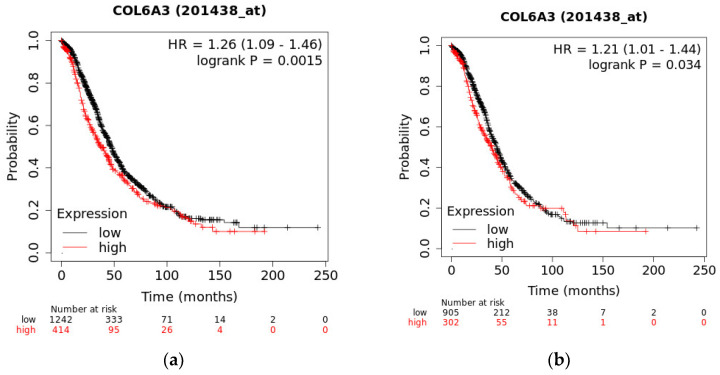
The overall survival regarding COL6A3 expression. The overall survival for high-expression COL6A3 in tissue was significantly higher than that of low expression in (**a**) all subtypes and (**b**) serous subtypes of EOC patients from TCGA and GEO data. The best cut-off determined by splitting patients into high- and low-expression groups was used as an auto-selection method, which was used to evaluate all possible cut-off values between the lower and upper quartiles of COL6A3 expression levels. The threshold that provided the best separation between the groups regarding survival outcomes was selected. This approach ensured that the cut-off point maximized the statistical power for detecting differences in survival between the high- and low-expression groups.

The OS for all subtypes and serous-subtype EOC patients from GEO (n = 1656) and TCGA (n = 1207) data with COL6A3 exhibited significantly worse outcomes in the high-expression group than in the low-expression group (*p* = 0.0015 and *p* = 0.034, respectively) (Figure 12).

### 2.11. GW4869 and Rampamycin Decreased Invasion Ability of EOC EXs

**Figure 13:**
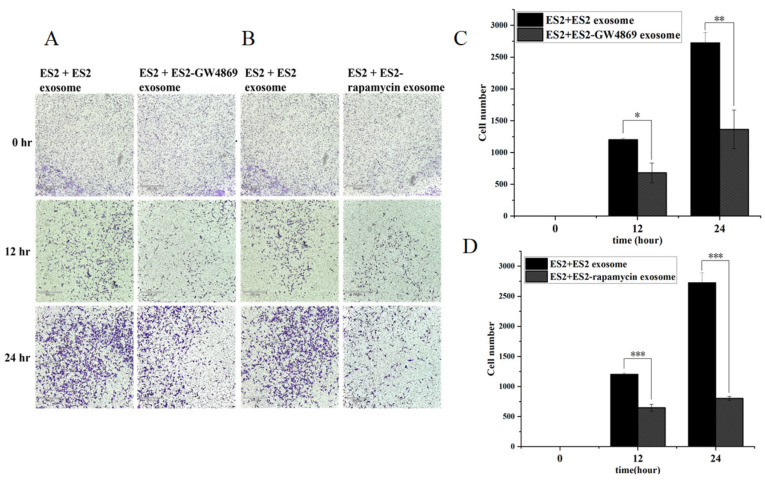
GW4869 and rapamycin decreased the invasion ability of EOC EXs. The invasion ability was inhibited more in ES2 with ES2-treated (**A**,**C**) GW4869 (* *p* < 0.05; ** *p* < 0.01, as determined using Student’s *t*-test) or (**B**,**D**) rapamycin exosomes than in ES2 with ES2 exosomes (*** *p* < 0.001, as determined using Student’s *t*-test).

We next examined if pharmacologic inhibition of exosome secretion could decrease the invasion ability of EOC cells. The EX biogenesis inhibitor GW4869 is the most widely used pharmacological agent for blocking EX generation, reducing EX release via nSMase inhibition, and reducing the number of EXs released. Additionally, inhibiting mTORC1 with rapamycin, a lysosome function enhancer and an autophagy inducer, can inhibit exosomal release. The invasion ability of ES2 cells treated with (A and C) GW4869 (10 nM) or (B and D) rapamycin (500 nM) was substantially lower than those treated with ES2 exosomes (*p* < 0.01 for GW4869 and *p* < 0.001 for rapamycin, respectively) (Figure 13).

## 3. Discussion

First, we found COL6A3 exosomes promoting tumor dissemination and metastasis in epithelial ovarian cancer. Genetic knockdown of COL6A3 or pharmacological inhibition of EX release can abolish invasion and metastasis in EOC. In this study, high expression of COL6A3 in EOC tissues associated with patients’ survival statuses was associated with a worse survival outcome than that for low expression based on the TCGA and GEO data. The expression of COL6A3 was significantly higher in the ovarian tumor and metastatic omentum tissues in the advanced stage than in the early stage in our EOC patients [21]. Importantly, COL6A3 was highly expressed in ES2 paclitaxel-resistant and ascites-derived MSC-OCSPC cells and EXs. Ascites displays aggressiveness and chemoresistance in ovarian cancer and leads to the dysregulation of lysosomal signaling, wherein lysosomes are critical for nutrient sensing and frequently associated with rapamycin complex 1 (mTORC1) [29]. Lysosomal signaling involves an energy demand for cancer cells in nutrient sensing [29]. Lysosomes are involved in the secretion of EX, and changes in lysosomal signaling and phenotype will also lead to changes in EX secretion [30], which has been implicated in cisplatin resistance. Autophagy can transport proteins through unconventional secretory pathways and carry cargo to lysosomes for the degradation of organelles via fusion with lysosomes to the plasma membrane and secrete cargo from the cell [31]. Previous studies have shown that cancer cells release more EXs than non-malignant cells, making the use of autophagy inhibitors to decrease EX secretion a new anticancer therapy strategy [32]. The EX biogenesis inhibitor GW4869 is the most widely used pharmacological agent for blocking EX generation, reducing EX release via nSMase inhibition, and reducing the number of EXs released. Inhibition of mTORC1 by rapamycin, a lysosome function enhancer and an autophagy inducer, also inhibits exosomal release [33].

Our previous studies show that COL6A3 regulates the CDK4/6-pRb and AKT-mTOR pathways and promotes EOC stemness, invasion, and metastasis [21,27]. The combination of everolimus (a mTOR inhibitor) and 5-aza-2-deoxycytidine (a demethylating agent) can effectively inhibit the production of ovarian clear-cell cancer stem-like or spheroid cells by inhibiting the COL6A3-AKT-mTOR pathway and exerting an anti-tumor effect [26]. An inhibitor against the mammalian target of rapamycin, temsirolimus, has been reported to be effective in patients with ovarian clear cell carcinoma [34]. However, the mTOR inhibitor everolimus and bevacizumab in recurrent ovarian cancer patients did not improve response compared to bevacizumab alone [35]. So far, mTOR inhibitor monotherapy or combination therapy for EOC has not yielded statistical results suitable for testing in phase III clinical trials. In this study, we confirmed that treatment with the EX inhibitor GW4869 or rapamycin abolished COL6A3-EXs and may have inhibited their aggressiveness in EOC. Limiting lysosomal exocytosis to reduce EX secretion may be an effective therapeutic strategy for reducing cancer cell invasiveness and chemoresistance [23]. Previous studies showed higher exosome-derived miR-200b and miR-200c concentrations in stage III–IV EOC patients with shortened OS [36]. In this study, we confirmed that treatment with the EX inhibitor GW4869 or rapamycin-abolished COL6A3-EXs may inhibit the aggressiveness of EOC. Limiting lysosomal exocytosis to reduce EX secretion may be an effective therapeutic strategy for reducing cancer cell invasiveness and chemoresistance [33].

Higher exosome-derived miR-200b and miR-200c concentrations in stage III-IV EOC patients are associated with shortened OS [36]. EXs from EOC patients have higher concentrations of TGFB1, melanoma-associated antigen 3 (MAGE3), and MAGE6 [37]. EXs from EOC patients also have higher concentrations of Claudin 4 associated with tumor stage and CA125 levels [38]. EXs isolated from EOC plasma samples had higher CD24 and EpCAM levels [39]. Furthermore, over 2000 proteins in EXs secreted from the OVCAR-3 and IGROV1 ovarian cancer cell lines have been identified and are involved in tumorigenesis and metastasis, creating the predictive potential of exosomal profiling [40]. However, more comprehensive clinical studies are needed to confirm the clinical value of this approach.

Cancer-associated fibroblasts (CAFs) can drive tumor proliferation, neo-vascularization, and invasion [41,42]. The reciprocal interactions between tumors and the stroma establish a local microenvironment that accelerates tumor progression [43]. ECM molecules signaling to stromal and cancer cells increase or decrease cancer progression. Type VI collagen is in the base membrane and interstitial matrix interface. During tumor progression, CAFs are the major players in dysregulated-collagen-based tumor fibrosis and the excessive deposition of collagen in tumors [44,45]. Collagen stiffens tissues through crosslinking and linearization, becoming fibroblast-derived stromal collagens, and directly correlates with poorer survival for cancer patients [46,47,48,49,50]. The exosomes from cancer cells, through reprogramming or signaling other cells, prolong tumor survival and promote metastasis [51,52]. However, exosomes from tumor microenvironments containing fibroblasts, mesothelial cells, adipocytes, and immune cells also affect cancer cells [53]. To this end, we carried out a pharmacological inhibition of nSMase2 and a genetic knockdown of COL6A3-decreasing exosomes. We confirmed that COL6A3 is secreted from EOC cells and tumor stroma via the exosomal pathway to affect EOC cells and ascites-derived MSC-OCSPCs. The results indicate that COL6A3 has established a premetastatic niche in the microenvironment.

Our data showed COL6A3 has expression in more aggressive ES2 derivatives, especially in ES2 paclitaxel-resistant cells and ascites-derived MSC-OCSPCs and the EXs derived from them, but not in less aggressive SKOV3 cells, ES2 knockdown COL6A3 (ES2/shCOL6A3), and thus-derived EXs. It is speculated that COL6A3 in more aggressive ES2 cells secreted from EXs remodels the ECM to affect ascites-derived stromal progenitor cells and establish a metastatic niche. However, COL6A3 expression in the differential responses of chemotherapy-naïve and relapsed EOC tissues will provide a better understanding of the potential of COL6 as a therapeutic target. COL6A3 and EXs may serve as novel diagnostic and prognostic biomarkers in cancer and contribute to clinical therapeutic applications in the future. COL6A3 secretion from the EX route is an uncovered field in EOC progression and metastasis. As COL6A3 is essential in facilitating tumor progression and metastasis, future studies targeting COL6A3 as a valuable biomarker for the early diagnosis of chemotherapy drug resistance, metastasis, recurrence, and the prediction of survival outcome by checking COL6A3 exosomes from the blood, ascites, or tissues of EOC patients are warranted, and the mediation of possible related signaling should be explored. COL6A3 research also holds promise for developing therapeutically targeting COL6-based conjugated antibodies or vaccines for EOC patients in the future.

## 4. Materials and Methods

### 4.1. Sample Collection

Ovarian cancer tissues and discarded ascites samples obtained from surgery or symptom relief from patients with primary or recurrent ovarian cancer were immediately taken to the laboratory for processing. In vitro isolation and culture of OCSPCs from ascites and cancerous tissues were performed as described previously [26]. Cell lines and cultures (ES2 and SKOV3) were obtained from the American Type Culture Collection (ATCC). Cells were maintained in a humidified atmosphere containing 5% CO_2_ at 37 °C and grown in McCoy’s 5A medium with 10% FBS. As previously described, we developed a paclitaxel-resistant ES2 cell line by continuously exposing cells to paclitaxel [27]. The final paclitaxel concentrations that induced paclitaxel-resistant subclones, called ES2TR, were 160 nM in size. 

### 4.2. Tumor Sphere Formation of Ovarian Cancer Stem-like Cells

ES2, ES2TR160, and ascites specimens isolated from EOC patients were cultured in tumor sphere (spheroid)-inducing conditions to induce tumor sphere formation. Briefly, cells were cultured in DMEM/F12 medium with 20 ng/mL of bFGF, 20 ng/mL of EGF, 10 ng/mL of IGF, and 2% B27 (Invitrogen, Carlsbad, CA, USA). Dissociated single cells (1 × 10^5^ cells/mL) were seeded into ultra-low attachment plates (Corning 3262, Pittston, PA, USA). After 7 days, we counted the spheres that had formed with an Olympus light microscope (Olympus, Tokyo, Japan). Then, tumor spheres obtained after 14 days were harvested and analyzed with flow cytometry. 

### 4.3. ExoQuick-TC™

Biofluid was collected and centrifuged at 3000× *g* for 15 min to remove cells and cell debris. The supernatant was transferred to a sterile vessel to add the appropriate volume of ExoQuick-TC to the biofluid. The well was mixed by inverting or flicking the tube, refrigerating overnight (at least 12 h) at +4 °C, and centrifuging the ExoQuick-TC/biofluid mixture at 1500× *g* for 30 min. After centrifugation, the supernatant was aspirated. The residual ExoQuick-TC solution was spun down by centrifugation at 1500× *g* for 5 min, and all fluid traces were removed by aspiration. Then, we resuspended the exosome pellet in 100–500 μL using sterile 1× PBS. 

### 4.4. Nanoparticle Tracking Analysis 

Purified exosomes were resuspended in 100 μL of 0.22 μm filtered PBS and analyzed using a NanoSight LM10 instrument (NanoSight, Salisbury, UK). The analysis was performed by applying a monochromatic 404 nm laser to dilute the exosomal preparation and measure the Brownian movements of each particle. Nanoparticle Tracking Analysis software version 2.3 was used to analyze 60 s videos of data collection to give the mean, median, and mode of vesicle size and concentration.

### 4.5. Extracellular Exosome (EX) Flow Cytometry Analysis

EXs were incubated with biotinylated antibody-coated beads in 500 μL of bead wash buffer (System Biosciences, Inc., Palo Alto, CA, USA) overnight in a 1.5 mL tube at 4 °C. After the binding step, beads were stained with either anti-CD9, anti-CD34, anti-CD63, anti-CD81, anti-CD105, anti-CD117, or anti-CD133 antibodies (BD Biosciences, Franklin Lakes, NJ, USA), which were either biotinylated, APC, FITC, Pacific Blue, PE, or PE-Cy7 conjugated. After antibody binding, beads were washed with bead wash buffer and recovered using a magnetic stand (optional, cat# EXOFLOW700A-1). When using a biotinylated antibody, a step involving incubation with streptavidin-FITC (System Biosciences, Inc.) was added, followed by EX stain buffer (System Biosciences, Inc.). Samples were analyzed using FACS LSRFortessa cytometers (BD Biosciences), and data were analyzed using BD FACSDiva™ Software v9.0 FACS Diva or FlowJo (BD Biosciences).

### 4.6. COL6A3 Knockdown and Overexpression

The COL6A3 knockdown in MSC-OCSPCs and ES2 cells and overexpression in SKOV3 cells were described previously [22]. 

### 4.7. LC-MS/MS Analysis

Protein digestion and dimethyl labeling of peptides were performed. The condition media were reduced with 10 mM of dithiothreitol, alkylated with 50 mM of iodoacetamide, and digested with Lys-C and trypsin. The digested peptides were labeled with isotopic formaldehyde (13CD_2_O, heavily labeled) and formaldehyde (CH_2_O, lightly labeled), respectively. Equal amounts of the heavily and lightly labeled peptides were mixed and desalted with StageTips with Empore TM SDB-CX disc membrane (3M, St. Paul, MN, USA).

NanoLC-MS/MS analyses. The peptides were analyzed using nanoLC-MS/MS on an online Dionex 3000 RSLC nanosystem (Thermo Fisher Scientific, Waltham, MA, USA) coupled with an LTQ Orbitrap XL mass spectrometer (Thermo Fisher Scientific). SpeedVac was used to dry the supernatant. Redissolved peptides with 0.5% acetic acid and 2% acetonitrile (ACN) and loaded onto an in-house-prepared 100 μm × 15 cm tip column were packed with 3 μm ReproSil-Pur 120 C18-AQ reverse-phase beads and eluted at a flow rate of 500 nL/min. The mobile phases used for nanoLC were 0.5% acetic acid in water (buffer A) and a mixture of 0.5% acetic acid and 80% ACN (buffer B). The LC gradient conditions were 5% to 40% buffer B in 60 min, 40% to 100% buffer B in 5 min, and 100% buffer B in 10 min. The LTQ Orbitrap XL system was operated in the positive ion mode, and full-scan MS spectra (*m*/*z* 300–1600) were acquired an Orbitrap analyzer with a resolution of 60,000 at *m*/*z* 400. Raw files from LC-MS/MS were analyzed using MaxQuant software v2.6.3.0. The differential expression levels were compared among different groups: group 1—ES2 cells and ES2 EXs versus ES2 cells; group 2—ES2 with ES2 TS EXs versus ES2 cells; and group 3—MSC-OCSPCs and ES2 EXs versus MSC-OCSPCs. The cutoff value was defined as a differential expression level >2.

### 4.8. Analysis of TCGA and GEO Data

We downloaded 372 TCGA OV RNA-Seq level 3 read count data (serous type) from the GDC Data Portal (https://portal.gdc.cancer.gov/ (accessed on 21 July 2024). The gene annotation file was used in GENCODE version 22 and obtained from GDC Reference Files (https://gdc.cancer.gov/about-data/gdc-data-processing/gdc-reference-files (accessed on 21 July 2024), which the TCGA program used. Clinical follow-up information was found in a PanCanAtlas publication (https://gdc.cancer.gov/about-data/publications/pancanatlas (accessed on 21 July 2024). We calculated the best cut-off by splitting patients into high- and low-expression groups, which was an autoselection process, and computed all possible cutoff values between the lower and upper quartiles, and the best-performing threshold was used as a cutoff. Microarray data from GEO and TCGA for all subtypes and RNA-seq data from the TCGA data set for all subtypes and serous types were used for survival analysis. The survival curve was plotted according to overall survival (OS) and progression-free survival (PFS) for 1656 and 1435 patients for GEO and TCGA data and 373 and 177 patients for TCGA data.

### 4.9. Invasion Experiments

For invasion assays, we used matrigel-coated transwell chambers (BD Biosciences, San Jose, CA, USA) that were inserted into 24-well cell culture plates. SKOV3 cells, ES2 cells, or MSC-OCSPCs (5 × 10^4^ cells in 0.2 mL of serum-free medium) were added to the upper chamber, and culture medium (McCoy’s 5A medium) was used in the lower chamber with serum-free conditions for the negative control, or containing 10% FBS for the positive control, or supplemented with culture medium (McCoy’s 5A medium) with serum-free conditions and treatment with EXs (30 μg) from SKOV3, SKOV3/COL6A3, ES2, ES2/shCOL6A3, ES2 TS, ES2TR, S2TR TS, CSPCs, MSC-OCSPCs/shCOL6A3, or ES2 cells treated with GW4869 or rampamycin cell extracts. Cells were cultured for 1, 3, or 7 days, and cells that invaded the inserts were fixed in methanol for 20 min, stained with crystal violet, and counted in three random microscope fields (Olympus BX3, Olympus, Tokyo, Japan) at a magnification of 40×, 100×, or 200×. 

### 4.10. Western Blot Analysis

Cells were lysed in phosphate-buffered saline (PBS) containing 1% Triton X-100 using an ultrasonic cell disruptor. Lysates were separated using SDS-PAGE (12.5%) and transferred to a polyvinylidene fluoride membrane (NEN). The membranes were blocked in blocking buffer (tris-buffered saline containing 0.2% Tween 20 and 1% I-block [NEN]) and incubated with polyclonal antibodies (Ab) separately for 1 h. A purified rabbit antihuman GAPDH polyclonal Ab (Santa Cruz Biotechnology, Inc., Dallas, TX, USA) was applied simultaneously to normalize the signals generated from the anti-COL6A3, CD9, and CD63 (Cell Signaling, Danvers, MA, USA). After washing, an alkaline phosphatase-conjugated anti-rabbit antibody (Vector Laboratories, Burlingame, CA, USA) was applied. The membranes were washed, and the bound Abs were visualized using nitroblue tetrazolium/5-bromo-4-chloro-3-indolyl phosphate chromogen.

### 4.11. In Vivo Animal Experiments and Tumor Imaging

Female null mice (BALB/cAnN.Cg-Foxn1nu/CrlNarl) were purchased from the National Animal Center (Taipei, Taiwan), and the Institutional Animal Care and Use Committee of Cathay General Hospital approved all experiments. In experiment 1, null mice at 5–7 weeks of age (5 mice/group) were injected intraperitoneally with luciferase-expressing SKOV3 cells, which displayed a less aggressive phenotype, and 10 μg of EXs from more aggressive ES2 cells or phosphate-buffered saline (PBS) were intraperitoneally injected twice weekly for 6 weeks. In experiment 2, null mice at 5–7 weeks of age (5 mice/group) were injected intraperitoneally with 1 × 10^6^ SKOV3/COL6A3 cells, which displayed a more aggressive phenotype, or 1 × 10^6^ less aggressive SKOV3 cells were injected into the peritoneal cavity. In experimental 3, 1 × 10^6^ SKOV3/COL6A3 cells or 1 × 10^6^ SKOV3 cells were administered intravenously into the tail vein of the mice. In experimental 4, SKOV3/COL6A3 cells were intravenously injected with 10 μg of EXs from SKOV3/COL6A3 cells or phosphate-buffered saline (PBS) twice weekly for up to 10 weeks to examine the tumor dissemination and growth. The body weight of mice was measured, recorded, and compared with the body change every week. The number and size of metastatic tumor nodules in mice were recorded and measured when mice were sacrificed. Disseminated tumor numbers were measured and counted using calipers, and volumes were calculated based on the modified ellipsoid formula (L × W × W/2). Tumor weights were measured following euthanasia at the endpoint. The histologic examination of tumor growth in the peritoneal cavity and lung was confirmed via H&E stains for diagnosis.

### 4.12. Statistical Analysis

Data were analyzed using SPSS 16.0 (SPSS Inc., Chicago, IL, USA). All numerical data are expressed as the mean ± SD from at least three experiments. Significant differences between the two groups were determined using the Student’s *t*-test, and important differences among more than two groups will be determined using a one-way ANOVA. Progression-free survival (PFS) and OS were calculated using the Kaplan–Meier method. Differences in survival curves were calculated using the log-rank test. *p* < 0.05 was considered statistically significant. *p* * represents *p* < 0.05, *p* ** represents *p* < 0.01, and *p* *** represents *p* < 0.001.

## Data Availability

Data is contained within the article and Supplementary Materials.

## Supplementary Materials

The following supporting information can be downloaded at: https://www.mdpi.com/article/10.3390/ijms25158121/s1.
