# Supplementary material for: COL6A3 Exosomes Promote Tumor Dissemination and Metastasis in Epithelial Ovarian Cancer
Source: Int J Mol Sci. 2024 Jul 25;25(15):8121. doi: 10.3390/ijms25158121 (PMC11311469; doi:10.3390/ijms25158121)

Fig-A Protein

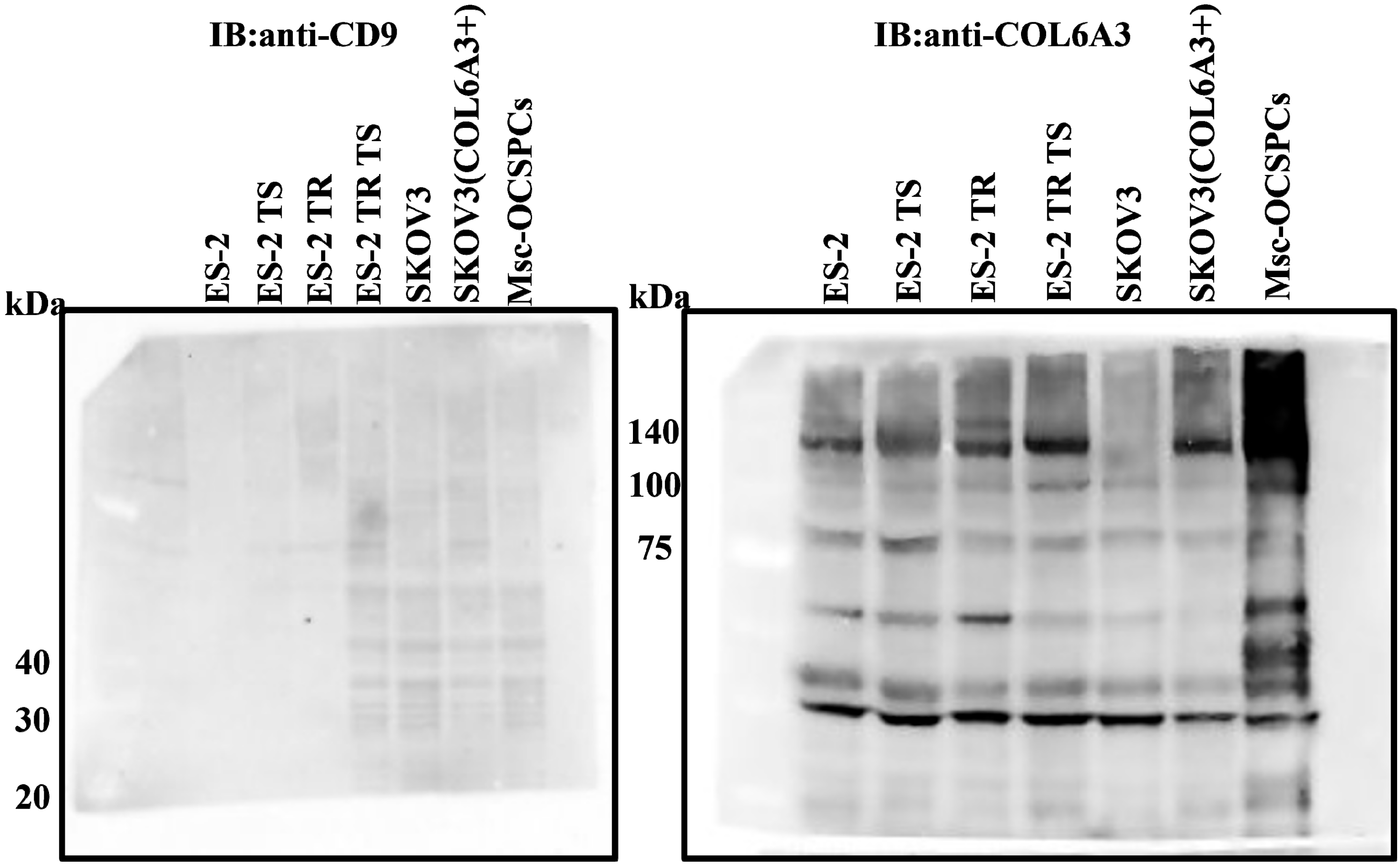

Fig-B Exosome

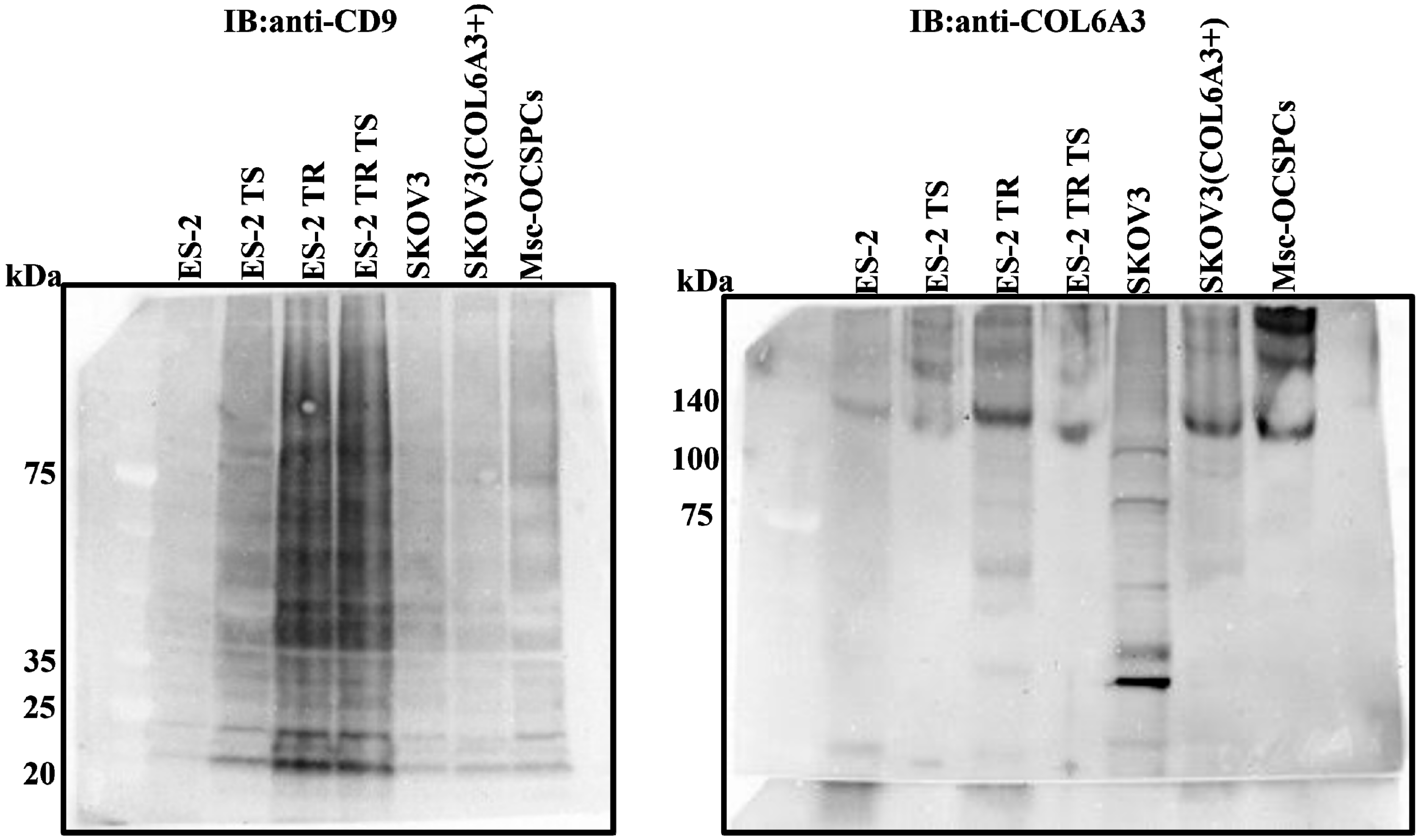

Fig-C Protein

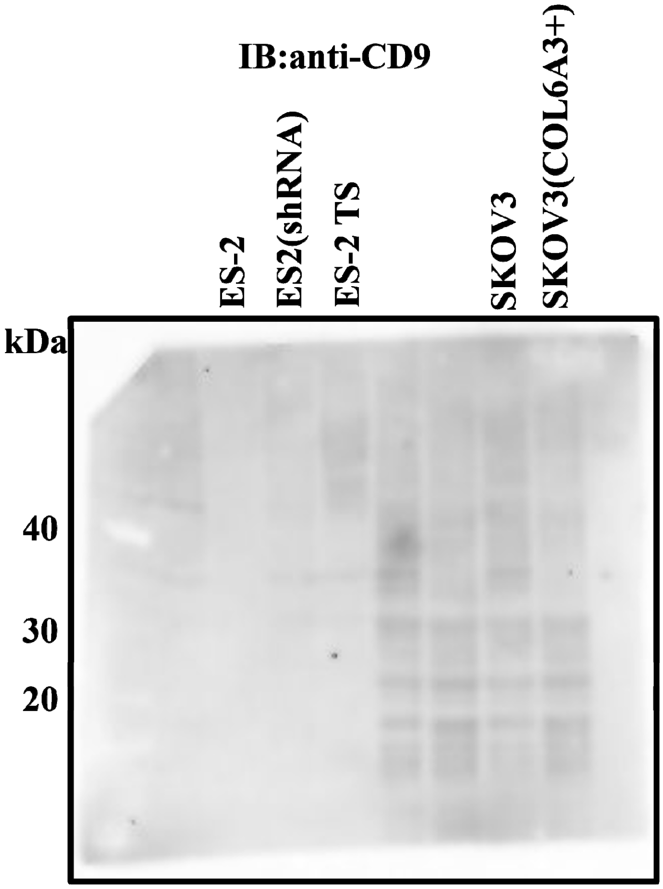

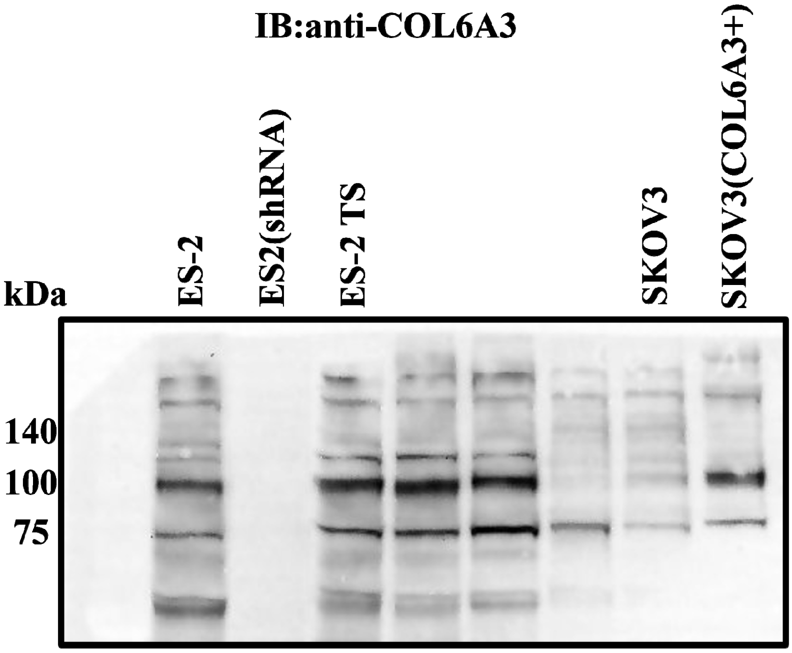

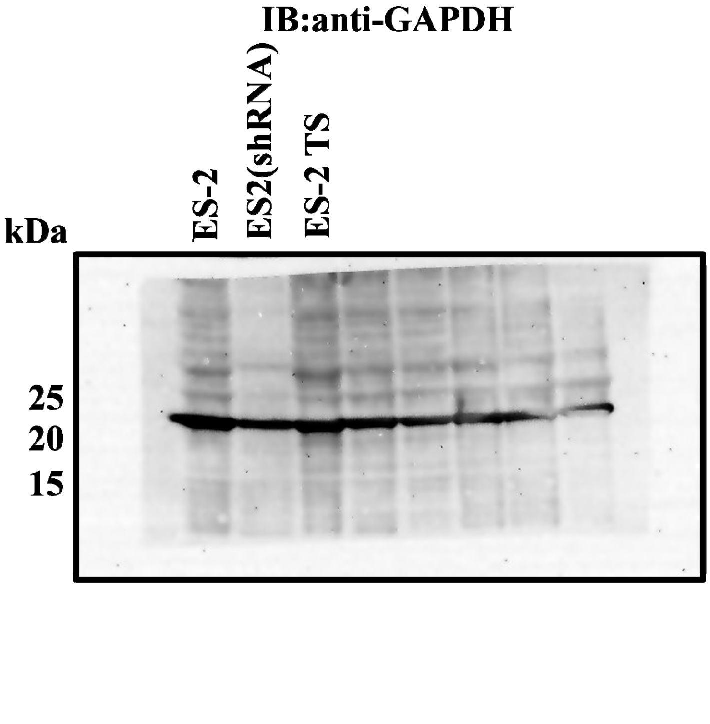

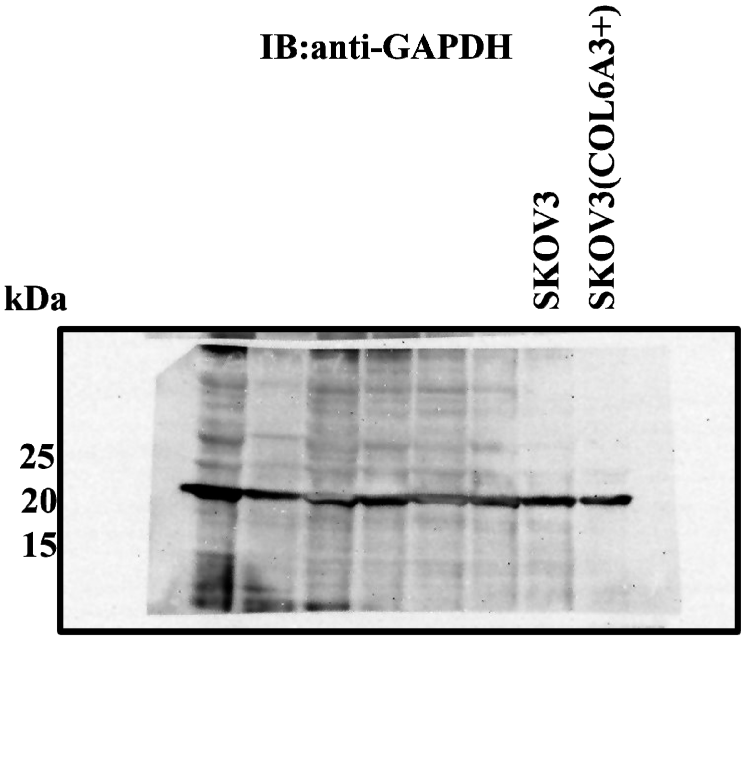

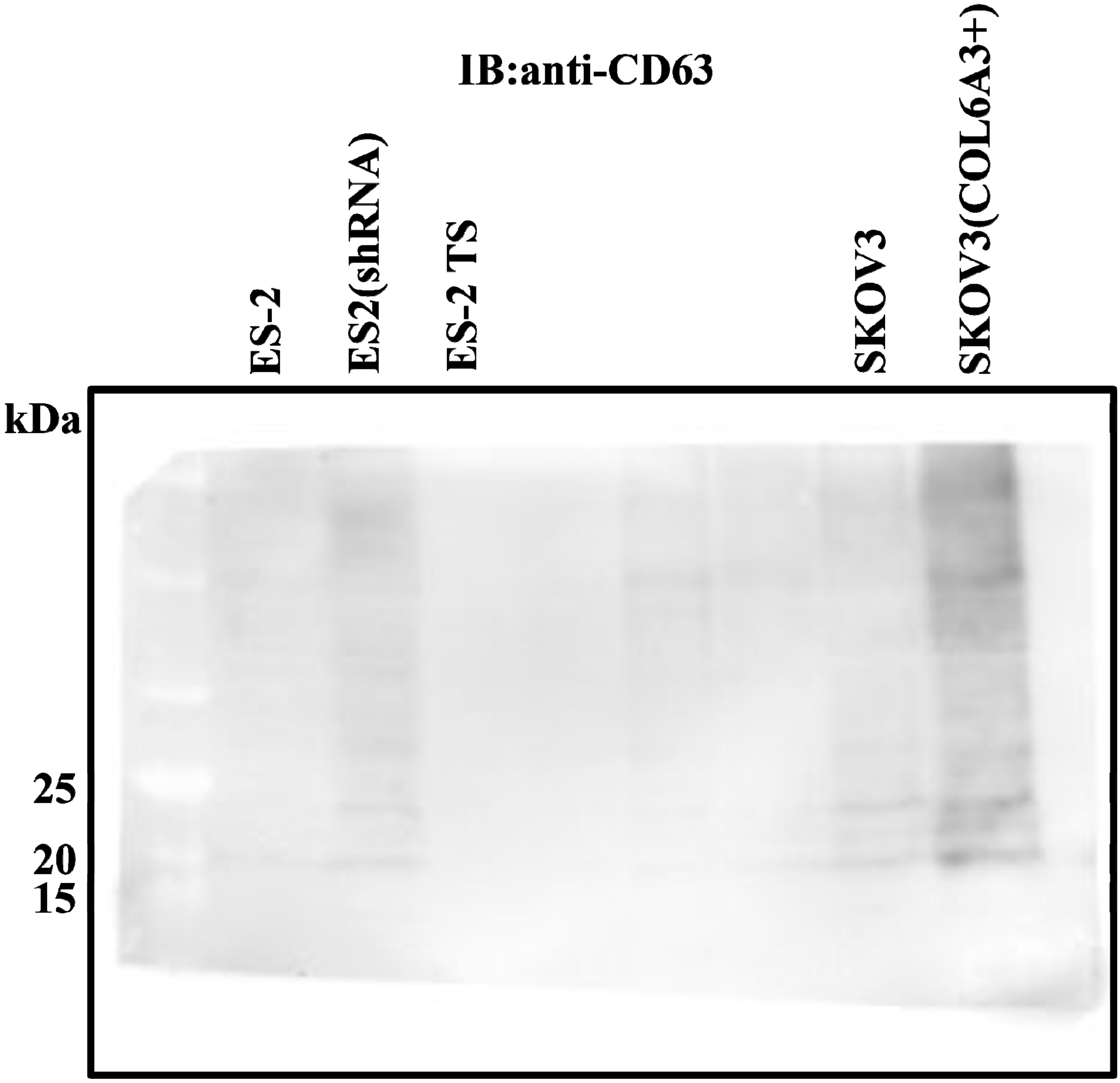

IB:anti-CD63
ES-2
ES2(shRNA)
ES-2 TS
SKOV3
SKOV3(COL6A3+)
kDa
25
20
15

Fig-C Exosome

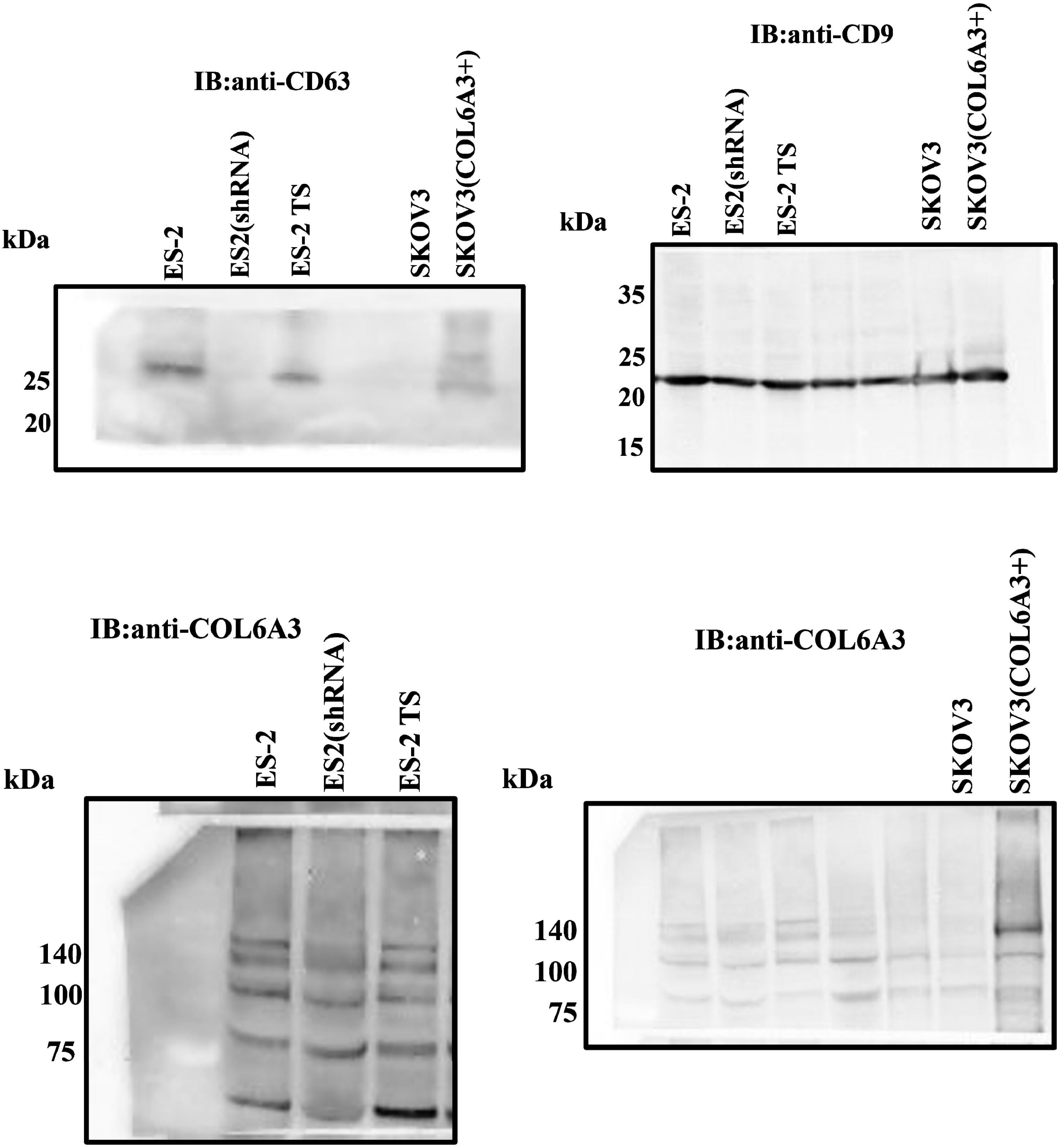

Supplement: Supplementary file 1 [file ijms-25-08121-s001.zip › ijms-3084663-supplementary.pdf]
